# CAPOX vs. FOLFOX for Colorectal Cancer—Real World Outcomes in Ontario, Canada

**DOI:** 10.3390/curroncol32080435

**Published:** 2025-07-31

**Authors:** Deepro Chowdhury, Gregory R. Pond, John R. Goffin

**Affiliations:** 1 Windsor Regional Hospital, Windsor, ON N8W 2X3, Canada; 2 Escarpment Cancer Research Institute, McMaster University and Hamilton Health Sciences, Hamilton, ON L8V 1C3, Canada; gpond@mcmaster.ca; 3 Department of Oncology, McMaster University, Juravinski Cancer Centre, 699 Concession St., Hamilton, ON L8V 5C2, Canada

**Keywords:** colorectal cancer, chemotherapy regimens, real-world outcomes, overall survival, CAPOX vs. FOLFOX

## Abstract

CAPOX and FOLFOX are widely used chemotherapy regimens for colorectal cancer (CRC). The superiority of one regimen over the other in a real-world setting (RWE) could have significant clinical implications given their common use, but such RWE is limited. This study analyzed provincial database records of 13,461 Canadian patients treated from 2005 to 2017. The primary outcomes were rates of Emergency Department visits and/or hospitalizations (ED/H) and overall survival (OS). CAPOX was used less frequently (8.4%) than FOLFOX (91.6%), often in older patients (*p* < 0.003 for Stage I–III; *p* < 0.001 for Stage IV). CAPOX recipients had shorter treatment durations (median 15 vs. 20 weeks, *p* = 0.002) and higher unadjusted ED/H rates (60.8% vs. 50.9%, *p* < 0.001), though this difference was nonsignificant on multivariate analysis (MVA) (HR 1.05 (0.92, 1.20), *p* = 0.466). Patients receiving CAPOX had worse OS than those on FOLFOX, (5-year OS 70.1% vs. 77.2% (*p* < 0.001) non-metastatic; 16.6% vs. 33.2% (*p* < 0.001) metastatic). MVA confirmed inferior OS with CAPOX (HR 1.42, *p* < 0.001). Other predictors of shorter OS included older age, male sex, comorbidities, rural residence, and lower income. This administrative data is at risk of bias but highlights the need for careful patient selection and informed treatment decision making.

## Simple Summary

In clinical trials, 5-fluorouracil (5-FU), leucovorin, and oxaliplatin (FOLFOX) and capecitabine and oxaliplatin (CAPOX) have been shown to be equally effective chemotherapy regimens for colorectal cancer. FOLFOX requires an inconvenient and failure-prone central venous access device (CVAD) to allow the infusion of 5-FU over a 46 h period every two weeks. While CAPOX removes the need for a CVAD, concerns about increased side effects have limited its use. We used a provincial administrative database to compare the efficacy and safety of FOLFOX and CAPOX and found that patients receiving CAPOX received somewhat shorter treatment but were not more likely to visit the ED or be hospitalized compared to those receiving FOLFOX. However, patients receiving CAPOX did have lower overall survival. While our findings are not definitive, they suggest that careful shared decision making is critical when deciding between these two chemotherapy regimens.

## 1. Introduction

FOLFOX (5-fluorouracil, leucovorin and oxaliplatin) chemotherapy is a mainstay of colon cancer treatment in both the adjuvant [1,2] and metastatic [3] settings. While effective, treatment involves significant patient burden as it requires the insertion of a central venous access device (CVAD) such as a peripherally inserted central catheter (PICC) for patients to receive a 46 h infusion of 5-fluorouracil every two weeks. The CVAD must be kept dry while bathing, regularly accessed, and can interfere with patients’ day-to-day functioning. Patients are also at risk of complications such as line infections and thromboses. One prospective study found that up to 25% of patients ultimately have complications from PICC lines, with 15% requiring PICC removal [4].

CAPOX is an alternate chemotherapy regimen which has been shown to be equivalent to FOLFOX in both the adjuvant [5] and metastatic [6,7] settings. Its main advantage is the use of capecitabine (Xeloda), a 5-FU equivalent that can be taken orally, which eliminates the need for a long-term CVAD. The regimens also differ in the oxaliplatin is given at a higher dose less frequently in the XELOX regimen 130 mg/m^2^ every 3 weeks instead of 85 mg/m^2^ every 2 weeks). Direct comparisons of these regimens have also, however, shown an increased incidence of hand-foot syndrome and high-grade diarrhea in patients receiving CAPOX [6,7,8]. As a result, despite evidence that the latter regimen is both cost-saving and more convenient for patients compared to FOLFOX [9], its use as adjuvant therapy prior to the publication of the 2018 IDEA meta-analysis—which showed that a 3-month course of adjuvant CAPOX was non-inferior to a 6-month course [10]—was limited [11].

Given the increased interest in CAPOX as a result of the IDEA findings and the differences in toxicity, the aim of the present study was to analyze the real-world impact of CAPOX versus FOLFOX chemotherapy in a Canadian setting prior to publication of IDEA. We used the ICES database, which links administrative data from several databases capturing essentially all patients in the province of Ontario, Canada. The province of Ontario has a population of almost 15 million people, allowing analyses using ICES to have sample sizes large enough to detect even small differences in outcomes that reflect real-world practice patterns.

## 2. Materials and Methods

The period examined was 2005 through 2017, which allowed us to compare similar duration treatments prior to publication of IDEA. Data collection for adjuvant patients began from the date of first Ontario Drug Benefit coverage of capecitabine for the adjuvant indication (31 May 2016), and for metastatic patients from the date of first start of funding in the metastatic setting (23 December 2008). Patient data was extracted from the ICES database, which includes diagnostic tests, tumor pathology, treatments received as well as medical event dates such as emergency department (ED) visits and hospitalizations for the entire provincial population, presently including over 15 million persons. Demographic data, including vital status, was derived from the provincial Registered Persons Database and Postal Code Conversion File. Chemotherapy treatment use was taken from the New Drug Funding Program and Cancer Activity Level Reporting database. Staging information was obtained through the Ontario Cancer Registry, while admission data was obtained through the National Ambulatory Care Reporting System (NACRS) as well as the Discharge Abstract Database (DAD). Social variables were collected using the Ontario Marginalization Index (ON-Marg), a province-wide data tool providing various demographic measures (e.g., economic, ethno-racial, etc.) to provide a quantifiable estimate of a patient’s overall socioeconomic marginalization based on their area of residence within the province. The primary outcomes of interest were overall survival in patients who received CAPOX or FOLFOX as well as rates of ED visits and/or hospitalizations while on treatment, comparing the two regimens. The study included patients who received either CAPOX or FOLFOX (with or without anti-EGFR, anti-VEGF and/or radiation therapy) in the adjuvant or metastatic setting for colon or rectal cancer. Patients were identified based on the International Classification of Diseases code (ICD-10) associated with their diagnosis (see Appendix A for a complete list of codes used to capture patients’ cancer diagnoses). Patients were excluded if they received any systemic treatment prior to their diagnosis of colorectal cancer, if they had surgery > 30 days pre-diagnosis, if they had no follow-up post-diagnosis, if they were Stage 0, and if they had a history of prior malignancy. Patients were considered to have received FOLFOX if they received 5-FU and oxaliplatin within 120 days of their first treatment; patients who received capecitabine and oxaliplatin within 120 days of their first treatment were ruled to have received CAPOX. The 120-day cut-off was used to maximize patient capture, since oxaliplatin is not given as treatment in CRC without the use of a fluoropyrimidine and adjuvant treatment beginning after that time would be unlikely. Patients were classified as having ED visits and/or hospitalizations while on treatment if they had either of those events, for any reason, from the first date of initiation of a chemotherapy regimen to 30 days after the last dose of that chemotherapy regimen. The last dose of chemotherapy was assigned if no further treatment was given within 6 weeks of that date, acknowledging that a few patients would be lost who resumed treatment while recognizing that incorporating chemotherapy holidays into the analysis was impractical.

Using SAS version 9.0 (SAS Institute, Cary, NC, USA) and R version 4.3.0, descriptive statistics were used to summarize patient demographic, cancer and treatment variables, along with outcomes. Univariable differences in baseline, cancer, treatment and outcome characteristics were explored using the Χ^2^ test, two-sample *t*-test or log-rank test, for categorical, continuous and time-to-event variables, respectively. Overall survival was defined from date of first treatment with CAPOX/FOLFOX until the date of death, with censoring of patients at the last date they were in contact with the Ontario health care system prior to the data cutoff date (31 March 2022). The Kaplan–Meier method was used to estimate overall survival. The effect of treatment type on frequency of ED/H, or on overall survival, was tested using logistic regression, or Cox proportional hazards regression analyses. All potential and available covariates were included in the regression model, and the effect of treatment was then assessed adjusting for all other factors. Statistical significance was defined at the alpha = 0.05 level and all tests and confidence intervals were two-sided. No adjustment for multiple testing was performed, but results were interpreted cautiously, understanding that multiple tests were conducted.

## 3. Results

**Figure 1:**
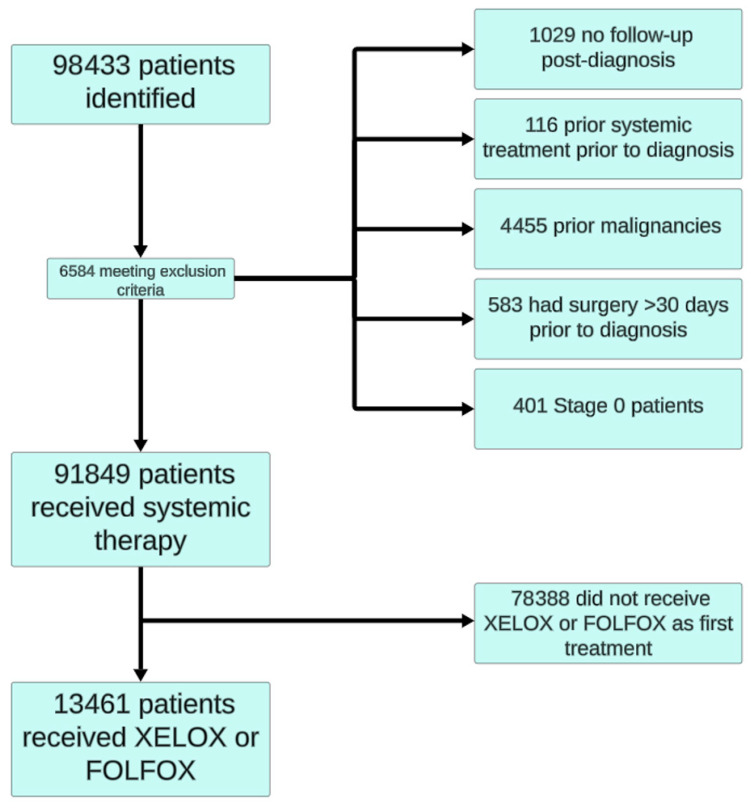
Consort flow diagram of patient selection.

A total of 98,433 patients were recorded as having been diagnosed with colorectal cancer in Ontario between 2005 and 2017, of whom 13,461 eligible individuals received either CAPOX or FOLFOX (see Figure 1).

### 3.1. Patient Characteristics and Treatment Data

Of 13,461 patients who received either CAPOX or FOLFOX, stage at diagnosis was known for 12,606; 2017 of these patients (16.0%) had Stage IV disease. Among all patients, FOLOX was administered to 91.6% (11,525) while 8.4% received CAPOX (1081) (see Table 1). Use of concurrent anti-EGFR therapy or VEGF-targeted therapy was rare, with only 23, 39 and 948 patients in total receiving cetuximab, panitumumab or bevacizumab, respectively. Patients treated with CAPOX were typically older (*p* < 0.003 for Stage I–III; *p* < 0.001 for Stage IV). Among patients with stage IV disease, men represented a higher proportion of those treated with FOLFOX than CAPOX (57.1% versus 49.1%, *p* = 0.029). A slightly higher proportion of patients receiving CAPOX had stage IV disease (214/1801, 19.8%) compared to FOLFOX (1803/11,525, 15.6%), although the use of CAPOX increased significantly among stage III patients in later years (*p* < 0.001). A slightly higher percentage of patients receiving CAPOX in the curative (Stage 1–3) setting were from rural areas compared to FOLFOX (18.5% versus 14.9%, *p* = 0.007). Patients who received CAPOX in the adjuvant setting underwent a median 15 weeks of treatment compared to 20 weeks with FOLFOX (*p* = 0.002) against a standard adjuvant duration of 24 weeks for either regimen. In the metastatic setting, median treatment duration was similar between the two groups (22.5 weeks of CAPOX versus 24 weeks of FOLFOX, *p* = 0.15) (see Table 2).

### 3.2. Treatment Outcomes

#### Emergency Department Visits and Hospitalizations

Patients treated with CAPOX had higher unadjusted rates of ED visits and/or hospitalizations while on treatment than those who received FOLFOX (60.8% versus 50.9%, *p* < 0.001) (see Table 2). In subset analyses, rates of ED visits, hospitalizations, or either combined remained higher in the CAPOX group for all groups except for those patients with metastatic.

In univariate analysis, receiving CAPOX as first systemic treatment was more likely to result in an ED visit or hospitalization (HR 1.54 (95% CI 1.36–1.74), *p* < 0.001), but in a multivariate analysis there was no significant difference between the regimens (HR 1.05 (95% CI 0.92–1.20), p = 0.47) (see Table 3). Rurality was an independent predictor of ED visits and/or hospitalizations (HR 1.30 (95% CI 1.17–1.43) in MVA, *p* < 0.001), as was Charlson score 2 (HR 1.23 (95% CI 1.00–1.50), *p* = 0.017) and treatment in a later year of diagnosis (HR 1.21/year (95% CI 1.20–1.22), *p* < 0.001). Variability in the ED visit or hospitalization risk was also observed according to disease site (*p* < 0.001), with an apparent increased risk in the rectosigmoid (HR 1.32 (95% CI 1.14–1.52)) and Rectum NOS (HR 2.45, 95% CI 2.16–2.76).

### 3.3. Overall Survival

Patients treated with CAPOX had lower rates of survival at 5 years than those who received FOLFOX among both non-metastatic (5-year OS 70.1% (66.6, 75.3) versus 77.2% (76.2, 78.1), *p* < 0.001) and metastatic (16.6% (11.0, 23.2) versus 33.2% (30.7, 35.6), <0.001) (see Table 2).

**Figure 2:**
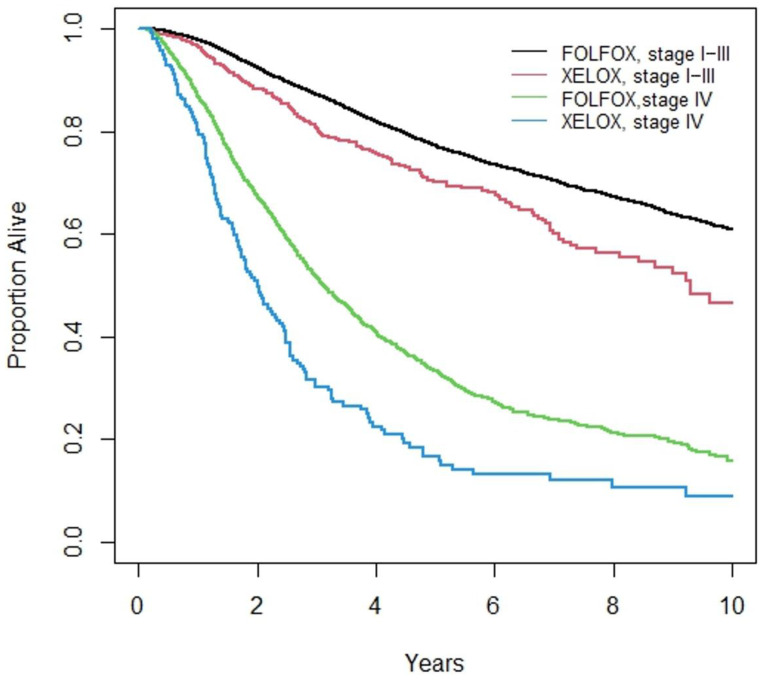
Survival curves of patients receiving CAPOX and FOLFOX (Stage I–III and Stage IV).

In multivariate analysis, patients receiving CAPOX as their first systemic treatment had higher risk of earlier death (HR 1.42 (95% CI 1.27–1.58), *p* < 0.001) (see Table 4). The survival difference was apparent in both the curative and metastatic populations, as seen in the Kaplan–Meier curves (see Figure 2).

Poorer survival was also associated with increasing age (*p* < 0.001), male sex (HR 1.08 (95% CI 1.01–1.15), *p* = 0.016), a non-zero Charlson score (*p* = 0.041), rural habitation (HR 1.15 (95% CI 1.06–1.24), *p* = 0.001), and lower income quintile (*p* = 0.04) (see Table 4).

Ontario public funding for capecitabine was only available for those 65 years of age and over, corresponding to the typically older population receiving CAPOX. A multivariate sensitivity analysis performed using only patients > 65 years old yielded results very similar to the overall analysis in terms of ED visits and/or hospitalization (HR 0.97 (95% CI 0.79–1.19)) as well as survival (HR 1.47 (95% CI 1.27–1.71)) (see Appendix B, Table A1 and Table A2).

## 4. Discussion

This report represents the largest analysis of real-world data (RWD) comparing the effectiveness and toxicity of FOLFOX and CAPOX. Our findings were somewhat unexpected, as ED visits and/or hospitalizations were not higher amongst patients treated with CAPOX but survival was worse. The first finding of note is that a comparatively small number of patients were treated with CAPOX in Ontario during the period studied. Of patients who received fluoropyrimidine doublet chemotherapy, 91.4% (11,525) received FOLFOX, while only 8.6% (1131) received CAPOX. Part of the discrepancy may have been a result of funding: provincial funding for capecitabine began in 2008 for the metastatic setting and in 2016 for the adjuvant indication, delaying its use outside of private drug insurance. Nevertheless, the 2017 cut-off date was thought to be useful in that it preceded the 2018 publication of the IDEA meta-analysis. This study, which suggested that 3 months of adjuvant CAPOX is non-inferior to 6 months in patients with low-risk (T3 and N1) disease, was thought likely to make it more difficult to compare treatments of similar length [2]. The difference in usage rates between the two regimens may also reflect a perception among many oncologists that CAPOX is a more poorly tolerated chemotherapy regimen despite the convenience of avoiding central venous access. However, despite this possible perception, patients receiving CAPOX were typically older; almost 20% of patients receiving CAPOX in the metastatic setting were 75 years old or older, compared to less than 10% of those receiving FOLFOX. Despite this age difference, however, overall co-morbidity between the CAPOX and FOLFOX cohorts, as represented by the Charlson scores for each population, was not significantly different. Additionally, a sensitivity analysis performed using only patients > 65 years old yielded almost identical outcomes (see Appendix B, Table A1 and Table A2).

Unadjusted rates of ED visits and/or hospitalizations were higher among patients who received CAPOX compared to FOLFOX, regardless of stage or time on treatment. However, when controlling for other factors the difference between the two regimens was not statistically significant. The similar ED/hospitalization rate in the two groups was counter to our expectation based on experience, although trial data is not informative. For example, in the NO16996 trial comparing FOLFOX and CAPOX in the first-line metastatic setting, rates of Grade 3 or higher diarrhea (based on CTCAE criteria; diarrhea severe enough to warrant hospitalization) were higher in the CAPOX arm compared to FOLFOX [6]. However, in that trial, overall grade 3/4 toxicity was similar in the two arms (78% vs. 72% for FOLFOX vs. CAPOX among the patients without bevacizumab), and hospitalization rates related to toxicity were not specified.

Prior real-world comparisons of the relative toxicity of these two regimens have yielded conflicting results. In 200 patients with metastatic CRC, Baqai et al. found that patients receiving FOLFOX experienced higher overall toxicity rates despite mucositis and hand-foot syndrome being more common among patients receiving CAPOX [8]. An analysis of SEER data found lower rates of health care utilization among patients who received CAPOX compared to FOLFOX, although the spectrum of claims used to determine this utilization was likely too limited to be definitive [12]. In contrast, three retrospective studies of CRC patients found that dose-limiting toxicity was higher in patients receiving CAPOX [13,14]. It is likely that many of the toxicities in these studies would not have led to emergency department assessment or hospitalization, making comparisons with our study difficult. Our study cannot account for dose reductions which may have diminished capecitabine-related toxicity. Prior retrospective studies suggest that doses of both drugs in CAPOX are reduced as compared with FOLFOX, while outcomes remain at least as good [13,14,15,16]. It must also be the case that some ED visits and/or hospitalizations in both arms were due to other medical conditions, disease complications, or disease progression, all of which would obscure the apparent toxicity of each regimen.

Of note, treatment durations between CAPOX and FOLFOX were similar in the metastatic (median 22.5 versus 24 weeks, *p* = 0.15) setting, but in the non-metastatic setting patients receiving CAPOX received less adjuvant treatment overall (median 20 versus 15 weeks in favor of FOLFOX, *p* = 0.002). This finding is consistent with prior data suggesting that fewer patients complete the intended length of adjuvant therapy with CAPOX compared to FOLFOX: for example, in a multicenter, retrospective analysis of 306 patients with Stage IIB and Stage III CRC from British Columbia, only 67% of patients treated with CAPOX completed the intended 24 weeks of therapy, compared to 82% of those who received FOLFOX [13]. The retrospective nature of our administrative dataset makes explaining this discrepancy in adjuvant treatment duration difficult, especially since adjusted rates of ED visits and/or hospitalizations were similar with each regimen regardless of disease stage. Patient preferences, baseline performance status and treatment-related toxicity not leading to ED visits or hospitalizations could all have contributed to the reduced treatment duration in the CAPOX cohort, but this data is not captured in the ICES database. Other significant predictors of ED visits and hospitalizations found in our study correlate with existing literature. Living in a rural location, for example, was associated with an increased risk of ED visits or hospitalizations (HR 1.30 on MVA), which was also demonstrated in a large population-based cohort of more than 650,000 cancer patients [17]. Patients with higher Charlson Comorbidity Index scores also had higher rates of ED visits and hospitalizations, in line with existing data [18].

In contrast to ED visits and hospitalizations, overall survival was significantly worse in patients who received CAPOX compared to FOLFOX. This unexpected finding was maintained after adjusting for other factors, including the greater age and more common stage IV disease in the CAPOX population (HR of 1.42 on multivariate analysis). This result contrasts with both randomized trial data as well as prior real-world analyses, which show comparable response rates [8] and overall survival rates [12,13,14,15]. Other factors in our study population associated with poorer overall survival such as increasing age, rural residence, comorbidities, cancer stage and sidedness are well-known adverse prognostic factors in CRC that have been described previously [19,20,21,22,23]. As would be expected, the survival contribution of factors such as performance status, lifestyle, smoking status at time of treatment, capecitabine adherence, and patient preferences were not available from the ICES administrative database and may have altered outcomes. Additional studies would be required in order to assess these potential differences using non-administrative datasets. The ICES database also does not track chemotherapy dose intensity, so it cannot be ruled out that more patients who received CAPOX required dose reductions. Although dose reductions may impair the overall efficacy of the regimen, some data suggest lower doses may still be effective [14,15,16,24].

Regional differences in drug tolerance could also have potentially affected outcomes. An analysis of 5-FU and capecitabine monotherapy within three phase III trials found that American patients were more likely to experience significant side effects with fluoropyrimidine therapy compared with their counterparts outside the United States. Interestingly, European clinicians are reported to routinely use the higher single-agent capecitabine (standard) 1250 mg/m^2^ twice daily dose in patients while US clinicians tend to use 1000 mg/m^2^ [25]. Dietary folate intake may partly explain this variation. Fluoropyrimidines require the presence of reduced folate for antitumor activity, and elevated serum folate levels predict greater toxicity in patients receiving capecitabine for treatment of colorectal cancer [26,27]. Canada, like the US, mandates the addition of folic acid to various grains, while this practice is relatively less common in Europe and most Asian countries, where patients are also generally more tolerant of fluoropyrimidines. Even so, it is not clear that there is a differential impact of folate levels on capecitabine versus 5-fluorouracil toxicity. Moreover, although unmeasured dose reductions in capecitabine in our study may have occurred, at least some prospective data suggest that intentional dose reduction does not negatively impact clinical outcomes [24]. Furthermore, limited comparison within clinical trials employing CAPOX among international populations do not show a clear difference in outcome by geographic region [5,6,7].

Our study has important limitations. The use of real-world data by definition means that the CAPOX and FOLFOX patient populations were not randomized, and as a result patients in the CAPOX cohort were more likely to be older, have metastatic disease and reside in rural areas. While these factors were incorporated in our MVA, other important unmeasured differences may exist, including patients’ smoking status, lifestyle factors, personal preferences, etc., that could have affected regimen usage and outcomes. Because of these limitations, our study cannot definitively show the superiority of one regimen over the other. In addition, the ICES database does not include dosing information and so other markers of potential toxicity such as dose reductions could not be captured. Reasons for differences in treatment duration cannot be determined from the database, including whether such dose reductions were for toxicity. We could not adjust for the potential impact of a shorter treatment duration on survival. As previously mentioned, the proportion of patients who received CAPOX was small (8.4%), although this number is almost identical to that found in the SEER study conducted by Satram-Hoang et al. [12]. Furthermore, although our study’s sample size (n = 12,656) is arguably representative of a general North American population, inferring similar results with respect to toxicity or survival outcomes to regions outside of Ontario is questionable. The fact that one outcome (overall survival) was observed to be statistically significant when multiple outcomes were evaluated implies that inferring a true differential effect on overall survival must be considered with caution, even though the actual level of significance observed was quite small (*p*-value < 0.001). Another limitation of administrative database studies such as ours is that data is collected algorithmically (rather than by individual chart review), and some data may have been misidentified. Finally, as patient and clinician decision making in choosing one regimen over the other and in utilizing ED/H were not available, any association between chemotherapy regimen and toxicity as measured by ED/H will be more difficult to discern.

## 5. Conclusions

In conclusion, our study suggests that CAPOX may not be associated with more ED visits or hospitalization than FOLFOX, offering reassurance regarding rates of severe toxicity. Conversely, our finding that CAPOX confers worse survival is a potential cause for concern, although caution is required when inferring results as mitigating factors cannot be identified. This study is a reminder of two seemingly contradictory facts: first, that the results of clinical trials do not necessarily translate into real-world outcomes; and second, that big data cannot replace the randomized clinical trial as a source of truth. In either case, while CAPOX remains a viable treatment for patients with CRC, patient selection and informed decision making remain paramount.

## Figures and Tables

**Table 1:** Demographic data of patients receiving CAPOX or FOLFOX.

Patient Characteristics		Stage 1–3			Stage 4		
		CAPOX	FOLFOX	*p*-Value	CAPOX	FOLFOX	*p*-Value
N		867	9722		214	1803	
**Age Groups**	N (%) 18–39	31 (3.6)	395 (4.1)	0.003	7 (3.3)	107 (5.9)	<0.001
	40–64	480 (55.4)	5760 (59.3)		100 (46.7)	1049 (58.2)	
	65–69	185 (21.3)	1812 (18.6)		39 (18.2)	280 (15.5)	
	70–74	100 (11.5)	1177 (12.1)		27 (12.6)	212 (11.8)	
	75–79	49 (5.7)	468 (4.8)		25 (11.7)	121 (6.7)	
	80+	22 (2.5)	110 (1.1)		16 (7.5)	34 (1.9)	
**Sex**	N (%) Male	495 (57.1)	5502 (56.6)	0.802	105 (49.1)	1030 (57.1)	0.029
**Year of Diagnosis**	2007	32 (3.7)	751 (7.7)	<0.001	26 (12.2)	171 (9.5)	0.303
	2008	43 (5.0)	955 (9.8)		23 (10.8)	176 (9.8)	
	2009	33 (3.8)	959 (9.9)		13 (6.1)	121 (6.7)	
	2010	35 (4.0)	958 (9.9)		13 (6.1)	111 (6.2)	
	2011	57 (5.4)	890 (9.2)		15 (7.0)	153 (8.5)	
	2012	53 (6.1)	960 (9.9)		15 (7.0)	200 (11.1)	
	2013	46 (5.3)	965 (9.9)		11 (5.1)	200 (11.1)	
	2014	64 (7.4)	910 (9.4)		13 (6.1)	206 (11.4)	
	2015	84 (9.7)	889 (9.1)		31 (14.5)	157 (8.7)	
	2016	228 (26.3)	761 (7.8)		28 (13.1)	160 (8.9)	
	2017	202 (23.3)	724 (7.5)		26 (12.2)	148 (8.2)	
**Rural**	N (%) Yes	160 (18.5)	1447 (14.9)	0.007	29 (13.6)	218 (12.1)	0.510
**Distance to Nearest RCC**	Median (IQR)	15.1 (6.0, 58.1)	12.6 (5.6, 45.0)	<0.001	10.5 (5.1, 34.8)	10.5 (5.1, 34.8)	0.393
**Charlson Score**	N (%) 0	204 (23.5)	2214 (22.8)	0.794	46 (21.5)	393 (21.8)	0.511
	1	34 (3.9)	375 (3.9)		8 (3.7)	52 (2.9)	
	2+	29 (3.3)	387 (4.0)		10 (4.7)	54 (3.0)	
	No admission	600 (69.2)	6746 (69.4)		150 (70.1)	1304 (72.3)	
**Hospital Type (for Surgery)**	Community	332 (71.4)	3388 (74.7)		86 (64.7)	648 (71.0)	
	Teaching	102 (21.9)	992 (21.9)		47 (35.3)	241 (26.4)	
**Instability Quintile**	1	155 (18.1)	1815 (18.9)	0.55	41 (19.4)	364 (20.3)	0.62
	2	170 (19.9)	2038 (21.2)		37 (17.5)	356 (19.9)	
	3	222 (25.9)	1975 (20.5)		41 (19.4)	306 (17.1)	
	4	157 (18.3)	1868 (19.4)		45 (21.3)	372 (20.8)	
	5	152 (17.8)	1935 (20.1)		47 (22.3)	394 (22.0)	
**Income Quintile**	1	153 (17.7)	1713 (17.7)	0.595	28 (13.1)	343 (19.1)	0.104
	2	176 (20.4)	1900 (19.6)		46 (21.5)	336 (18.7)	
	3	179 (20.7)	1995 (20.6)		42 (19.6)	366 (20.4)	
	4	182 (21.0)	2026 (20.9)		46 (21.5)	378 (21.0)	
	5	175 (20.2)	2058 (21.2)		52 (24.3)	375 (20.9)	
**Deprivation Quintile**	1	170 (19.9)	1979 (20.6)	0.92	55 (26.1)	384 (21.4)	0.20
	2	183 (21.4)	1995 (20.7)		38 (18.0)	353 (19.7)	
	3	180 (21.0)	1974 (20.5)		45 (21.3)	373 (20.8)	
	4	166 (19.4)	1942 (20.2)		37 (17.5)	334 (18.6)	
	5	157 (18.3)	1741 (18.1)		36 (17.1)	348 (19.4)	
**Dependency Quintile**	1	121 (14.1)	1790 (18.6)	<0.001	42 (19.9)	338 (18.9)	0.58
	2	156 (18.2)	1873 (19.5)		33 (15.6)	394 (22.0)	
	3	164 (19.2)	1850 (19.2)		46 (21.8)	335 (18.7)	
	4	197 (23.0)	1871 (19.4)		44 (20.9)	321 (17.9)	
	5	218 (25.5)	2247 (23.3)		46 (21.8)	404 (22.5)	
**Ethnicity Quintile**	1	225 (26.3)	2103 (21.8)	<0.001	47 (22.3)	352 (19.6)	0.22
	2	193 (22.6)	1910 (19.8)		39 (18.5)	327 (18.3)	
	3	168 (19.6)	1765 (18.3)		38 (18.0)	355 (19.8)	
	4	131 (15.3)	1835 (19.1)		53 (25.1)	360 (20.1)	
	5	139 (16.2)	2018 (21.0)		34 (16.1)	398 (22.2)	

CAPOX: capecitabine–oxaliplatin; FOLFOX: 5-fluorouracil, leucovorin and oxaliplatin; RCC: regional cancer center; IQR: interquartile range.

**Table 2:** Treatment outcomes among all patients receiving CAPOX or FOLFOX.

	Category		CAPOX	FOLFOX	*p*-Value
**N**			1081	11,525	
**5-year Overall Survival (%)**	Stage I–III		70.1 (66.6, 75.3)	77.2 (76.2, 78.1)	<0.001
	Stage IV		16.6 (11.0, 23.2)	33.2 (30.7, 35.6)	<0.001
**Median Weeks on Treatment (Interquartile Range, Highest Number)**	Stage I–III		15 (6–21), 30	20 (14–24), 28	0.002
	Stage IV		22.5 (12–27), 207	24 (16–28), 372	0.15
**ED Visits—N (%)**	On treatment		461 (42.7)	3713 (32.2)	<0.001
	Within 60 days		281 (26.0)	2025 (17.6)	<0.001
**Hospitalizations—N (%)**	On treatment		435 (40.2)	3918 (34.0)	<0.001
	Within 60 days		164 (15.2)	1023 (8.9)	<0.001
**ED Visits or hospitalization while on-treatment—N (%)**	All patients	Within 60 days	359 (33.2)	2608 (22.6)	<0.001
		On–treatment	657 (60.8)	5870 (50.9)	<0.001
	Stage I–III	Within 60 days	283 (32.6)	2189 (21.9)	<0.001
		On–treatment	522 (60.2)	4688 (48.2)	<0.001
	Stage IV	Within 60 days	76 (35.5)	480 (26.6)	0.008
		On–treatment	135 (63.1)	1182 (65.6)	0.49

CAPOX: capecitabine–oxaliplatin; FOLFOX: 5-fluorouracil, leucovorin and oxaliplatin; ED: emergency department.

**Table 3:** Univariate and multivariate analyses—ED visits and hospitalizations.

Factor	Comparator	Univariate		Multivariate	
		Odds Ratio (95% CI)	*p*-Value	Odds Ratio (95% CI)	*p*-Value
**Age Groups**	18–39	Reference	0.3	Reference	0.354
	40–64	0.80 (0.67, 0.94)		0.84 (0.70, 1.00)	
	65–69	0.81 (0.68, 0.98)		0.86 (0.71, 1.04)	
	70–74	0.81 (0.67, 0.99)		0.91 (0.74, 1.11)	
	75–79	0.79 (0.63, 0.99)		0.91 (0.72, 1.15)	
	80–84	0.82 (0.59, 1.16)		0.84 (0.59, 1.21)	
	85+	0.66 (0.28, 1.54)		0.57 (0.23, 1.42)	
**Sex**	Male vs. Female	0.99 (0.93, 1.06)	0.83	0.99 (0.93, 1.07)	0.877
**Year of Diagnosis**	/year	1.20 (1.19, 1.22)	<0.001	1.21 (1.20, 1.22)	<0.001
**Income Quintile**	1	Reference	0.16	Reference	0.824
	2	0.92 (0.82, 1.03)		0.98 (0.87, 1.10)	
	3	0.90 (0.81, 1.01)		0.95 (0.84, 1.06)	
	4	0.91 (0.81, 1.01)		0.97 (0.87, 1.09)	
	5	0.87 (0.78, 0.97)		0.94 (0.84, 1.06)	
**Rural**	Yes vs. No	1.23 (1.12, 1.35)	<0.001	1.30 (1.17, 1.43)	<0.001
**Charlson Score**	0	Reference	0.057	Reference	0.017
	1	0.97 (0.80, 1.17)		0.86 (0.70, 1.05)	
	2+	1.29 (1.07, 1.56)		1.23 (1.00, 1.50)	
	No Admission ⱡ	1.04 (0.96, 1.13)		0.94 (0.86, 1.03)	
**Site of Primary Lesion**	Cecum	Reference	<0.001	Reference	<0.001
	Ascending colon	0.84 (0.74, 0.96)		0.87 (0.76, 1.00)	
	Hepatic flexure	0.87 (0.69, 1.10)		0.97 (0.76, 1.24)	
	Transverse colon	0.99 (0.83, 1.18)		0.99 (0.82, 1.19)	
	Splenic flexure	0.83 (0.66, 1.04)		0.85 (0.67, 1.08)	
	Descending colon	0.97 (0.81, 1.16)		0.97 (0.80, 1.18)	
	Sigmoid colon	0.86 (0.77, 0.96)		0.90 (0.80, 1.02)	
	Overlapping region	1.87 (0.45, 7.85)		2.93 (0.67, 12.86)	
	Colon NOS	1.45 (0.77, 2.75)		1.12 (0.57, 2.21)	
	Rectosigmoid junction	1.29 (1.13, 1.47)		1.32 (1.14, 1.52)	
	Rectum NOS	2.34 (2.09, 2.63)		2.45 (2.16, 2.76)	
**Stage**	1	Reference	<0.001	Reference	<0.001
	2	0.58 (0.41, 0.80)		0.62 (0.44, 0.87)	
	3	0.47 (0.34, 0.64)		0.46 (0.33, 0.63)	
	4	0.95 (0.68, 1.32)		0.99 (0.70, 1.39)	
	Unknown	0.27 (0.19, 0.38)		0.60 (0.42, 0.86)	
**1st Systemic Treatment Received**	CAPOX vs. FOLFOX	1.54 (1.36, 1.74)	<0.001	1.05 (0.92, 1.20)	0.466

CAPOX: capecitabine–oxaliplatin; FOLFOX: 5-fluorouracil, leucovorin and oxaliplatin; NOS: not otherwise specified.

**Table 4:** Univariate and multivariate analyses—overall survival.

Factor	Comparator	Univariate		Multivariate	
		Odds Ratio (95% CI)	*p*-Value	Odds Ratio (95% CI)	*p*-Value
**Age Groups**	18–39	Reference	<0.001	Reference	<0.001
	40–64	0.94 (0.80, 1.10)		0.97 (0.83, 1.14)	
	65–69	1.09 (0.92, 1.29)		1.12 (0.94, 1.32)	
	70–74	1.46 (1.23, 1.73)		1.51 (1.27, 1.80)	
	75–79	1.99 (1.66, 2.39)		1.93 (1.60, 2.32)	
	80–84	2.69 (2.11, 3.44)		2.36 (1.84, 3.02)	
	85+	4.84 (2.90, 8.09)		3.93 (2.34, 6.58)	
**Sex**	Male vs. Female	1.08 (1.02, 1.15)	0.011	1.08 (1.01, 1.15)	0.016
**Year of Diagnosis**	/year	1.05 (1.04, 1.07)	<0.001	1.05 (1.04, 1.06)	<0.001
**Income Quintile**	1	Reference	0.015	Reference	0.04
	2	1.14 (1.04, 1.26)		0.97 (0.88, 1.06)	
	3	1.09 (0.99, 1.20)		0.97 (0.88, 1.07)	
	4	1.08 (0.98, 1.18)		0.89 (0.81, 0.98)	
	5	0.99 (0.90, 1.09)		0.89 (0.80, 0.97)	
**Rural**	Yes vs. No	1.08 (1.00, 1.17)	0.065	1.15 (1.06, 1.24)	0.001
**Charlson Score**	0	Reference	0.006	Reference	0.041
	1	1.20 (1.03, 1.41)		1.16 (0.99, 1.36)	
	2+	1.10 (0.93, 1.28)		1.09 (0.93, 1.28)	
	No Admission ⱡ	0.96 (0.89, 1.02)		0.96 (0.90, 1.03)	
**Site of Primary Lesion**	Cecum	Reference	<0.001	Reference	<0.001
	Ascending colon	0.95 (0.85, 1.06)		0.95 (0.85, 1.06)	
	Hepatic flexure	0.95 (0.79, 1.14)		0.98 (0.82, 1.18)	
	Transverse colon	1.01 (0.88, 1.16)		0.99 (0.86, 1.14)	
	Splenic flexure	0.86 (0.72, 1.04)		0.81 (0.67, 0.97)	
	Descending colon	0.72 (0.61, 0.85)		0.70 (0.60, 0.83)	
	Sigmoid colon	0.74 (0.67, 0.81)		0.72 (0.65, 0.79)	
	Overlapping region	1.01 (0.38, 2.70)		1.01 (0.38, 2.69)	
	Colon NOS	1.92 (1.25, 2.97)		1.22 (0.79, 1.89)	
	Rectosigmoid junction	0.79 (0.70, 0.88)		0.72 (0.64, 0.81)	
	Rectum NOS	0.72 (0.65, 0.79)		0.73 (0.66, 0.81)	
**Stage**	1	Reference	<0.001	Reference	<0.001
	2	0.95 (0.66, 1.36)		0.99 (0.69, 1.42)	
	3	1.35 (0.96, 1.91)		1.36 (0.97, 1.92)	
	4	5.50 (3.89, 7.77)		5.45 (3.85, 7.70)	
	Unknown	1.66 (1.16, 2.37)		1.99 (1.39, 2.85)	
**1st Systemic Treatment Received**	CAPOX vs. FOLFOX	1.69 (1.52, 1.88)	<0.001	1.42 (1.27, 1.58)	<0.001

CAPOX: capecitabine–oxaliplatin; FOLFOX: 5-fluorouracil, leucovorin and oxaliplatin.

## Data Availability

All data used in this research is available upon request.

## Appendix A. Data Definitions

### Appendix A.1. Cancer Variables

- Cancer diagnosis: colon or rectal cancer diagnosis by ICD-10 codes C18, C19, C20.
  ○ ICD C18.0–C18.9 (colon), C19.9 (rectosigmoid junction), or C20.9 (rectum).
- Cancer diagnosis date = date of first OHIP billing date for colon or rectal cancer plus an ‘any time’ positive Ontario Cancer Registry recording.
- Cancer stage: I to IV per the Ontario Cancer Registry.

### Appendix A.2. Treatment Variables

- First Chemotherapy Date = First date of administration within 365 days of diagnosis.
- Chemotherapy regimens:
  ○ FOLFOX = 5-fluorouracil administered within 365 days of diagnosis with oxaliplatin prescribed at any point during the treatment period (since oxaliplatin is only used in combination with a fluoropyrimidine).
  ○ CAPOX = a prescription for capecitabine is filled with oxaliplatin infused within 7 days.

### Appendix A.3. Treatment Variable Data Interpretation

- Curative (adjuvant) vs. Palliative vs. Relapse chemotherapy definitions:
  ○ Curative (adjuvant) chemotherapy = a 5-FU, capecitabine, FOLFOX, or CAPOX regimen begun within 4 months after a curative surgical procedure and having stage II or III colorectal cancer.
  ○ Relapse chemotherapy = prior receipt of “curative chemotherapy” according to the above definition, followed by another, different, colorectal cancer chemotherapy regimen starting at least 12 months after the surgical date.
  ○ Palliative chemotherapy = colorectal chemotherapy regimen given for Stage IV disease by registry.
  ○ Number of cycles = Number of oxaliplatin doses given prior to Day 200 post-operatively (in Stage II–III patients) or over patient’s lifetime (in Stage IV patients).

### Appendix A.4. Outcome Variables

Definitions:

- Hospitalization during treatment = hospitalization for any reason from first date of a chemotherapy regimen to 30 days after last dose of a chemotherapy regimen.
- Death—Y/N. Age at death or last follow-up. Last follow-up is otherwise defined as the date of the last OHIP billing or hospital discharge for the individual.
  ○ Death during chemotherapy = death from first date of a chemotherapy regimen to 30 days after last dose of that same chemotherapy regimen.

## Appendix B. Supplemental Analyses

**Table A1:** Univariate and multivariate analyses—ED visits and hospitalizations. Patients > 65 only.

Factor	Comparator	Univariate		Multivariate	
		Odds Ratio (95% CI)	*p*-Value	Odds Ratio (95% CI)	*p*-Value
**Age Groups**	65–69	Reference	0.98	Reference	0.67
	70–74	1.00 (0.88, 1.14)		1.09 (0.95, 1.24)	
	75–79	0.97 (0.82, 1.15)		1.09 (0.91, 1.31)	
	80–84	1.01 (0.75, 1.38)		1.06 (0.76, 1.47)	
	85+	0.81 (0.35, 1.87)		0.75 (0.31, 1.82)	
**Sex**	Male vs. Female	1.06 (0.94, 1.18)	0.34	0.98 (0.87, 1.11)	0.80
**Year of Diagnosis**	/year	1.19 (1.17, 1.21)	<0.001	1.20 (1.18, 1.22)	<0.001
**Income Quintile**	1	Reference	0.16	Reference	0.48
	2	0.92 (0.82, 1.03)		1.09 (0.90, 1.32)	
	3	0.90 (0.81, 1.01)		1.15 (0.95, 1.39)	
	4	0.91 (0.81, 1.01)		0.99 (0.82, 1.20)	
	5	0.87 (0.78, 0.97)		1.10 (0.91, 1.33)	
**Rural**	Yes vs. No	1.32 (1.14, 1.53)	<0.001	1.38 (1.17, 1.61)	<0.001
**Charlson Score**	0	Reference	0.27	Reference	0.16
	1	1.01 (0.77, 1.32)		0.89 (0.67, 1.19)	
	2+	1.30 (0.99, 1.69)		1.25 (0.94, 1.66)	
	No Admission ⱡ	1.07 (0.94, 1.21)		0.94 (0.81, 1.08)	
**Site of Primary Lesion**	Cecum	Reference	<0.001	Reference	<0.001
	Ascending colon	0.89 (0.74, 1.07)		0.97 (0.79, 1.18)	
	Hepatic flexure	1.00 (0.71, 1.39)		1.07 (0.76, 1.52)	
	Transverse colon	1.09 (0.84, 1.42)		1.11 (0.84, 1.46)	
	Splenic flexure	0.65 (0.45, 0.94)		0.67 (0.45, 0.98)	
	Descending colon	0.95 (0.70, 1.29)		0.97 (0.70, 1.35)	
	Sigmoid colon	0.94 (0.79, 1.12)		1.02 (0.85, 1.23)	
	Overlapping region	0.73 (0.12, 4.40)		1.27 (0.20, 8.07)	
	Colon NOS	2.20 (0.55, 8.83)		2.85 (0.65, 12.47)	
	Rectosigmoid junction	1.30 (1.04, 1.61)		1.36 (1.08, 1.71)	
	Rectum NOS	2.14 (1.78, 2.58)		2.27 (1.86, 2.77)	
**Stage**	1	Reference	<0.001	Reference	<0.001
	2	0.30 (0.15, 0.60)		0.38 (0.19, 0.77)	
	3	0.24 (0.13, 0.47)		0.31 (0.15, 0.61)	
	4	0.43 (0.22, 0.84)		0.58 (0.29, 1.17)	
	Unknown	0.13 (0.06, 0.26)		0.34 (0.16, 0.71)	
**1st Systemic Treatment Received**	CAPOX vs. FOLFOX	1.41 (1.17, 1.70)	<0.001	0.97 (0.79, 1.19)	0.78

**Table A2:** Univariate and multivariate analyses—overall survival. Patients > 65 only.

Factor	Comparator	Univariate		Multivariate	
		Odds Ratio (95% CI)	*p*-Value	Odds Ratio (95% CI)	*p*-Value
**Age Groups**	65–69	Reference	<0.001	Reference	<0.001
	70–74	1.34 (1.21, 1.49)		1.34 (1.21, 1.48)	
	75–79	1.82 (1.61, 2.06)		1.72 (1.52, 1.95)	
	80–84	2.49 (2.03, 3.05)		2.14 (1.74, 2.64)	
	85+	4.46 (2.72, 7.32)		3.51 (2.12, 5.82)	
**Sex**	Male vs. Female	1.06 (0.97, 1.16)	0.23	1.09 (0.99, 1.19)	0.084
**Year of Diagnosis**	/year	1.06 (1.04, 1.08)	<0.001	1.05 (1.03, 1.07)	<0.001
**Income Quintile**	1	Reference	0.30	Reference	0.16
	2	0.99 (0.86, 1.14)		1.00 (0.87, 1.16)	
	3	1.00 (0.88, 1.15)		0.85 (0.74, 0.99)	
	4	0.87 (0.76, 1.01)		0.98 (0.85, 1.13)	
	5	0.95 (0.83, 1.09)		0.95 (0.83, 1.10)	
**Rural**	Yes vs. No	1.09 (0.97, 1.22)	0.16	1.17 (1.04, 1.32)	0.008
**Charlson Score**	0	Reference	0.042	Reference	0.23
	1	1.24 (1.01, 1.52)		1.17 (0.96, 1.44)	
	2+	0.97 (0.79, 1.20)		1.07 (0.86, 1.32)	
	No Admission ⱡ	0.94 (0.85, 1.05)		0.97 (0.88, 1.08)	
**Site of Primary Lesion**	Cecum	Reference	0.008	Reference	0.004
	Ascending colon	0.98 (0.85, 1.14)		0.95 (0.82, 1.10)	
	Hepatic flexure	0.91 (0.70, 1.18)		0.85 (0.65, 1.11)	
	Transverse colon	1.05 (0.86, 1.29)		1.00 (0.82, 1.23)	
	Splenic flexure	1.11 (0.86, 1.44)		1.03 (0.79, 1.33)	
	Descending colon	0.77 (0.60, 0.99)		0.72 (0.56, 0.93)	
	Sigmoid colon	0.82 (0.72, 0.95)		0.79 (0.69, 0.91)	
	Overlapping region	0.69 (0.17, 2.78)		0.99 (0.25, 4.01)	
	Colon NOS	0.86 (0.32, 2.31)		0.67 (0.25, 1.80)	
	Rectosigmoid junction	0.88 (0.74, 1.05)		0.83 (0.69, 0.99)	
	Rectum NOS	0.78 (0.68, 0.91)		0.76 (0.66 (0.89)	
**Stage**	1	Reference	<0.001	Reference	<0.001
	2	0.82 (0.50, 1.35)		0.88 (0.53, 1.45)	
	3	0.93 (0.57, 1.50)		0.95 (0.58, 1.53)	
	4	3.42 (2.11, 5.55)		3.36 (2.07, 5.47)	
	Unknown	1.00 (0.61, 1.65)		1.21 (0.72, 2.01)	
**1st Systemic Treatment Received**	CAPOX vs. FOLFOX	1.77 (1.53, 2.04)	<0.001	1.47 (1.27, 1.71)	<0.001
